# Outcomes associated with asymptomatic bacteriuria management in elderly patients hospitalized with a ground-level fall

**DOI:** 10.1017/ash.2024.493

**Published:** 2025-03-03

**Authors:** Katelin A. Everitt, Margaret Baldwin, Nick Tinker, Ku’ulei Stuhr, David S. Morris, John J. Veillette

**Affiliations:** 1 Department of Pharmacy, Utah Valley Hospital, Provo, UT, USA; 2 Department of Pharmacy, Intermountain Medical Center, Murray, UT, USA; 3 Trauma Services, Intermountain Medical Center, Murray, UT, USA; 4 Infectious Diseases Telehealth Service, Intermountain Medical Center, Murray, UT, USA; 5 Emergency Department, Intermountain Medical Center, Murray, UT, USA

## Abstract

Data are lacking to guide management of asymptomatic bacteriuria (ASB) in elderly patients with a fall. Comparing treated versus non-treated patients, we identified clear harm and no benefit from antibiotic treatment. Our data support IDSA recommendations to withhold antibiotics in elderly patients with ASB and evaluate alternative causes of falls.

## Background

Asymptomatic bacteriuria (ASB), a positive urine culture (UC) without symptoms of a urinary tract infection (UTI), affects up to 50% of elderly adults.^1^ Randomized trial data support Infectious Disease Society of America (IDSA) recommendations against antibiotic treatment of ASB in numerous patient populations. However, data are lacking to support IDSA’s recommendation among elderly patients with a fall and ASB to observe and assess for other causes rather than treat with antibiotics. Overprescription of antibiotics for these patients leads to adverse events and delayed diagnosis of other conditions causing falls (e.g. dehydration, stroke, medication effects),^2,3^ while undertreatment might pose a theoretical risk of future UTI. Several studies have described ASB overtreatment in elderly patients, but comparative outcomes data are needed in elderly patients with a fall, which often prompts urine testing and treatment.^2–8^ Herein, we describe outcomes associated with ASB management in elderly patients hospitalized for a ground-level fall (GLF) at an urban 503-bed, level 1 trauma center.

## Methods

Patients ≥ 65 years of age hospitalized for a GLF requiring trauma service consultation between 1/2018 and 8/2023 were identified for screening. We then excluded patients electronically who had systemic signs of infection (temperature >38^o^C, systolic blood pressure <90 mmHg, or white blood cell count >12,000/µL), no urinalysis (UA) obtained, negative UA (negative leukocyte esterase, nitrites, bacteria, and <5 white blood cells per high-powered field),^9^ or who died during admission. The UA, rather than UC, was used because antibiotics are often prescribed for UA results before the UC is available. Patients were then excluded during manual chart review if they had cystitis or pyelonephritis symptoms (dysuria, urgency, frequency, suprapubic pain, flank pain, costovertebral angle tenderness), received antibiotics prior to admission, or received antibiotics for non-UTI indications. Patients with only non-localizing symptoms (e.g. abdominal pain) were classified as ASB if a clear non-UTI diagnosis was documented as the cause. If no clear non-UTI diagnosis was recorded in patients with non-localizing symptoms, the patient was classified as unknown and excluded from the analysis. Finally, we excluded patients who might experience UTI without typical symptoms (e.g. dementia, chronic urinary catheter, anuria, urologic cancer, or neurogenic bladder) and those with possible UTI based on imaging (e.g. bladder wall thickening). The remaining patients with ASB were split into two cohorts: those treated (inpatient and/or prescription upon discharge) versus not treated with antibiotics.

Demographics, comorbidities, hospital course, antibiotic treatment, laboratory values, vital signs, trauma activation level, Injury Severity Score, Charlson Comorbidity Index, and all-cause mortality and readmissions were obtained electronically, whereas manual review was used to assess UTI symptoms and the most likely cause of any readmissions.

The primary outcome was 90-day all-cause readmission, with description of subcategories (e.g. readmissions from antibiotic effects or new or worsening UTI). Secondary outcomes included provider variability in antibiotic prescribing for ASB, antibiotic treatment days and associated costs, 90-day *Clostridioides difficile* infection (CDI), all-cause 90-day mortality, and 90-day incidence of bacteremia (with the same organism as index UC). We used a two-tailed Fisher’s Exact test for categorical data and descriptive statistics for continuous data. The Institutional Review Board exempted this study as quality improvement (1052544), and we adhered to the STROBE guidelines for reporting observational studies.^10^

## Results

**Figure 1.**
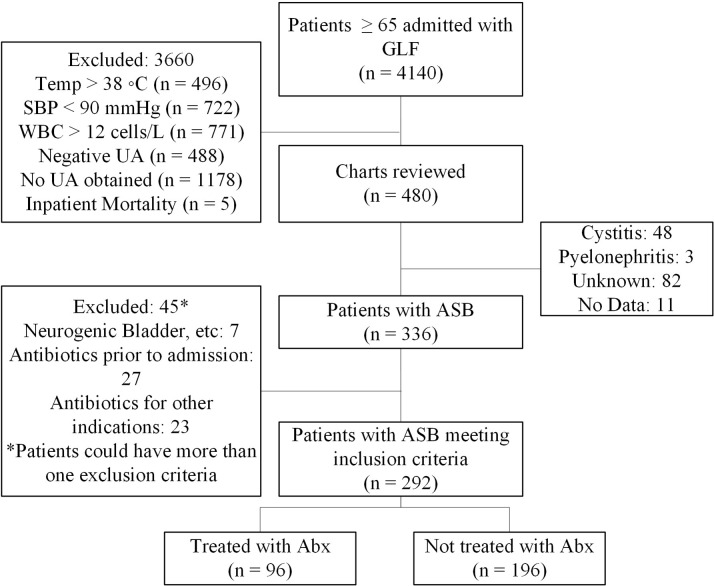
Inclusion/Exclusion Criteria. Abx, antibiotics, ASB, asymptomatic bacteriuria, GLF, ground-level fall, SBP, systolic blood pressure, Temp, temperature, UA, urinalysis, WBC, white blood cell count.

Of 4,140 patients screened, 480 (11.6%) were manually reviewed, and 292 (7.1%) met inclusion criteria: 96/292 (32.9%) were treated with antibiotics, and 196/292 (67.1%) were not (Fig. 1). Baseline characteristics were similar between groups, except the rates of abnormal UA findings and admission by the trauma service were numerically higher in the treated group (Table 1). All-cause readmission rates were not significantly different between the treated and untreated groups [14 (14.5%) versus 37 (18.9%), respectively, *P* = 0.36], nor were UTI-related readmission rates [4 (4.2%) versus 6 (3.1%), *P* = 0.43]. The treated group had 1 patient (1%) readmitted due to renal failure from trimethoprim-sulfamethoxazole and 1 patient (1%) who developed outpatient CDI following inpatient treatment with meropenem. There was no significant difference between the treated and untreated groups in 90-day all-cause mortality [13 (13.5%) versus 16 (8.2%), *P* = 0.16] or subsequent bacteremia with the index UC organism [1 (1%) versus 0 (0%), *P* = 0.33].

**Table 1.** Baseline characteristics

Characteristic^a^	Treated (n = 96)	Untreated (n = 196)
**Hospital Admission Year**		
2018 – 2020	52 (54.2)	110 (56.1)
2021 – 2023	44 (45.8)	86 (43.9)
**Median Age (IQR), years**	83 (77–89)	83 (76–89)
Age ≥ 80 years	61 (63.5)	123 (62.8)
**Male**	14 (14.6)	28 (14.3)
**Median Injury Severity Score (IQR)**	9 (4–10)	9 (5–10)
**Median Charlson Comorbidity Index (IQR)**	9 (7–12)	8 (6–12)
**Positive Component of UA** ^b^		
Leukocyte Esterase	86 (89.6)	103 (52.6)
Nitrites	33 (34.4)	26 (13.3)
Bacteria	68 (70.8)	113 (57.5)
White Blood Cells ≥ 5	90 (93.8)	103 (52.6)
**Symptoms**		
Not Reported in Chart	63 (65.6)	152 (77.6)
Non-Localizing^c^, With Explanation	3 (3.1)	11 (5.6)
Patient Denied UTI Symptoms	32 (33.3)	42 (21.4)
**Admitting Team Specialty**		
General Surgery	61 (63.5)	109 (55.6)
Trauma service	38 (39.6)	66 (33.7)
Internal Medicine	29 (30.2)	64 (32.7)
Neurology	1 (1.0)	3 (1.5)
Orthopedic Surgery	5 (5.2)	20 (10.2)
**Trauma Activation Level at Admission** ^d^		
Trauma I	--	1 (0.5)
Trauma II	5 (5.2)	20 (10.2)
Trauma III	21 (21.9)	35 (17.9)

Abx, Antibiotics; ADE, Adverse Event; SD, Standard Deviation; UA, Urinalysis; UTI, Urinary Tract Infection.

a Represented as number (%) unless otherwise stated.

b Patients could have more than one positive component of the UA.

c Non-localizing symptoms included abdominal pain, nausea, vomiting, altered mental status, or weakness. Several patients had non-localizing symptoms (with clear non-UTI cause) and denied UTI symptoms (n = 2 in the treated group, n = 9 in the untreated group).

d The remaining patients were admitted without meeting criteria for trauma activation, but the trauma service was still consulted.

Of the admitting providers for whom at least 5 patients were included [n = 11/77 (14%) of all providers in the study), the rate of antibiotic prescribing for ASB ranged from 14 to 58%. In the treated group, 379 days of antibiotics were given for ASB resulting in excess cost of USD $1,008.46 to the hospital, which included 313 inpatient antibiotic doses and 331 doses given after discharge (total of 644 doses). Using an average antibiotic duration of 3.9 days and cost of $2.66 per day per patient in the treated group, the untreated group avoided 764 days of antibiotic therapy and saved approximately $2,033.30.

## Discussion

Treating ASB with antibiotics did not benefit elderly patients hospitalized with a GLF, and there was clear evidence of harm. Our findings align with Boerckel et al.,^6^ who did not find antibiotic treatment to benefit hospitalized elderly patients with ASB and altered mental status (AMS). Notably in that study, patients for whom antibiotics were withheld had higher rates of alternative diagnoses explaining AMS. This treatment approach (i.e. avoiding anchoring on urine tests) should also be applied to GLFs, which have many causes besides UTI. Our findings support the IDSA recommendation in elderly patients with a fall to prioritize observation and investigation for other causes over antibiotic treatment of ASB.^1^

Our study was strengthened by detailed chart review and conservative exclusion criteria to identify elderly GLF patients with the highest likelihood of having ASB. These data identify low-hanging fruit for stewardship clinicians to intervene and educate providers (especially those with higher prescribing rates) to withhold antibiotics in this patient population. While most urine screening and treatment were driven by GLFs, we observed some overlap with surgeons treating ASB as peri-operative prophylaxis; hence, antibiotic review in this population might lead to multiple intervention and education opportunities.

Our study had numerous limitations. Only 7% of screened patients met inclusion criteria, which limits generalizability and implies more uncertainty in the excluded patients (some of whom might benefit from antibiotic treatment). Our retrospective data are limited by potential selection bias and reliance on documentation in the electronic medical record. Chart reviews were performed by a single clinical pharmacist, who was not blinded to the study objectives. Confounders that were not captured (e.g. undocumented UTI symptoms or pharmacist intervention to stop antibiotics) could have influenced treatment, and we could not account for readmissions to hospitals outside our healthcare system. We did not identify significant differences in readmissions, but larger studies with propensity weighted analysis could strengthen our findings. We could not capture other harms of antibiotics besides readmissions (e.g. development of antibiotic resistance). Finally, a 2021–2022 system initiative to educate providers on non-treatment of ASB could have introduced bias by altering provider documentation of UTI symptoms; however, this was likely addressed via our conservative exclusion criteria.

In summary, among elderly patients with ASB admitted for a GLF, we found evidence of harm and no clinical benefit from antibiotic treatment, which supports IDSA recommendations to observe, investigate other causes for falls, and withhold antibiotics.
